# Copy number gain of granulin-epithelin precursor (GEP) at chromosome 17q21 associates with overexpression in human liver cancer

**DOI:** 10.1186/s12885-015-1294-x

**Published:** 2015-04-11

**Authors:** Man Kuen Yung, Kwok Wai Lo, Chi Wai Yip, Grace TY Chung, Carol YK Tong, Phyllis FY Cheung, Tan To Cheung, Ronnie TP Poon, Samuel So, Sheung Tat Fan, Siu Tim Cheung

**Affiliations:** 1 Department of Surgery, The University of Hong Kong, Hong Kong, China; 2 Centre for Cancer Research, The University of Hong Kong, Hong Kong, China; 3 State Key Laboratory for Liver Research, The University of Hong Kong, Hong Kong, China; 4 Department of Anatomical and Cellular Pathology, The Chinese University of Hong Kong, Hong Kong, China; 5 Department of Surgery, Queen Mary Hospital, Hong Kong, China; 6 Department of Surgery, Stanford University, Stanford, USA; 7 Department of Surgery, The University of Hong Kong, L9-55, Laboratory Block, Faculty of Medicine Building, 21 Sassoon Road, Hong Kong, China

**Keywords:** Granulin-epithelin precursor, Chromosome gain, Liver cancer

## Abstract

**Background:**

Granulin-epithelin precursor (GEP), a secretory growth factor, demonstrated overexpression in various human cancers, however, mechanism remain elusive. Primary liver cancer, hepatocellular carcinoma (HCC), ranks the second in cancer-related death globally. GEP controlled growth, invasion, metastasis and chemo-resistance in liver cancer. Noted that GEP gene locates at 17q21 and the region has been frequently reported to be amplified in subset of HCC. The study aims to investigate if copy number gain would associate with GEP overexpression.

**Methods:**

Quantitative Microsatellite Analysis (QuMA) was used to quantify the GEP DNA copy number, and fluorescent in situ hybridization (FISH) was performed to consolidate the amplification status. GEP gene copy number, mRNA expression level and clinico-pathological features were analyzed.

**Results:**

GEP DNA copy number determined by QuMA corroborated well with the FISH data, and the gene copy number correlated with the expression levels (n = 60, *r* = 0.331, *P* = 0.010). Gain of GEP copy number was observed in 20% (12/60) HCC and associated with hepatitis B virus infection status (*P* = 0.015). In HCC with increased GEP copy number, tight association between GEP DNA and mRNA levels were observed (n = 12, *r* = 0.664, *P* = 0.019).

**Conclusions:**

Gain of the GEP gene copy number was observed in 20% HCC and the frequency comparable to literatures reported on the chromosome region 17q. Increased gene copy number contributed to GEP overexpression in subset of HCC.

**Electronic supplementary material:**

The online version of this article (doi:10.1186/s12885-015-1294-x) contains supplementary material, which is available to authorized users.

## Background

Granulin-epithelin precursor (GEP) contributes in multiple vital biological processes and its alias partly indicates its function, including progranulin, proepithelin, acrogranin, and PC cell-derived growth factor. GEP is a pluripotent growth factor important in fetal development, neuronal cell survival, wound healing and tumorigenesis [1-4]. Over-expression of GEP has been reported in a number of human cancers including breast, prostate, and ovary cancers [5-7]. In addition to its classical well-known biological function on growth regulation, GEP has recently been shown to control chemo-resistance [8-10]. Nonetheless, the mechanism of GEP over-expression remains elusive.

The incidence of primary liver cancer, hepatocellular carcinoma (HCC), has been increasing globally in the past two decades. HCC is the second most frequent cause of cancer-related death worldwide [11]. Major risk factors for HCC development include infections with hepatitis B virus (HBV) and hepatitis C virus (HCV), alcoholic liver diseases and fatty liver diseases. In China, with endemic HBV infection, HCC is the second leading cause of cancer death [12]. In Western countries, HCC incidence is increasing steadily [11]. Treatment of HCC remains a challenge, as curative partial hepatectomy and liver transplantation are only applicable for early stage patients [13]. However, with limited surveillance, and early stage HCC usually asymptomatic, the majority of HCC patients are diagnosed at advanced stages. Prognosis of advanced stage HCC is poor with overall survival rate less than 5%. Treatment options for advanced HCC are limited, systemic chemotherapy and hormone therapy have not been effective [14]. The research effort should continue to comprehend hepatocarcinogenesis. GEP has shown over-expression in HCC with functional roles on growth, invasion, tumorigenicity and cancer stem cell properties [8,9,15]. In addition, GEP has demonstrated the potential to serve as therapeutic target [16-18]. Noted the GEP gene locus at chromosome 17q21 and the region has been frequently reported with copy number gain in HCC [19,20]. In this study, we investigated the GEP DNA copy number, and analyzed the association with gene expression levels and clinico-pathological features.

## Methods

### Clinical specimens

Sixty patients underwent curative partial hepatectomy for hepatocellular carcinoma (HCC) between September 2002 and July 2005 at Queen Mary Hospital, Hong Kong, were recruited with informed consent. Clinico-pathological data were prospectively collected. Ten blood samples from healthy individuals were recruited and served as control with informed consent. The study protocol was approved by the Institutional Review Board of the University of Hong Kong / Hospital Authority Hong Kong West Cluster (HKU/HA HKW IRB).

### Quantitative microsatellite analysis (QuMA)

Copy number of GEP gene was measured by Quantitative Microsatellite Analysis (QuMA) as described using quantitative real-time PCR amplification [21]. Chromosome 3 consistently showed stable copy number in HCC [19,20,22] and thus microsatellite D3S1609 was used as a reference locus. Copy Number Assay for GEP and D3S1609 were ready-made reagents (Applied Biosystems, Foster City, CA). The number of PCR cycles (CT) required for the signal intensities to exceed a threshold just above background was calculated for the test and reference reactions. CT values were determined for test and reference reactions in each sample, averaged, and subtracted to obtain deltaCT (dCT) [dCT = CT (test locus) – CT (reference locus)]. dCT values were measured for each unknown sample [dCT (test DNA)] and for samples from ten unrelated healthy individuals (calibrator) [average dCT (calibrator DNA)]. Relative copy number at each locus in the test sample was then calculated as described [21]:

$$ \mathrm{Relative}\kern0.5em \mathrm{D}\mathrm{N}\mathrm{A}\kern0.5em \mathrm{copy}\kern0.5em \mathrm{number}=\mathrm{N}\times {\left(1+\mathrm{E}\right)}^{\hbox{-} \mathrm{ddCT}} $$

where ddCT = dCT (test DNA) – average dCT (calibrator DNA), and E = PCR efficiency. The primers had showed PCR efficiencies >95% (Additional file 1: Figure S1), and N = 2 for diploid normal individuals, and for simplicity, relative DNA copy number = 2 × 2^-ddCT^. Tolerance interval (TI) was calculated to determine if the test sample in the QuMA measurement was significantly different from the mean of measurements made on samples from the healthy individuals [21]:

$$ \mathrm{T}\mathrm{I}=\mathrm{N}\pm \mathrm{S}\mathrm{D}\times 3.38 $$

where SD was the standard deviation and 3.38 was the two-sided tolerance limiting factor for the measurements on healthy samples, and N = 2 for diploid status. Measurements outside this range were considered significantly different from normal.

### Fluorescence in situ hybridization (FISH)

FISH analysis was performed to confirm the copy number status in paraffin-embedded HCC tissues as described [23]. Two primary HCCs were investigated by FISH based on histology assessment among the samples with DNA copy number gain by QuMA. Two BAC clones RP11-436 J4 and RP11-52 N13 flanking GEP gene located at 17q21 were labelled with Spectrum Green (Molecular Probes, Life Technologies). The centromeric probes at chromosome 3 (pAE0.68) and chromosome 17 (pEZ17-4) were labelled with Spectrum Orange (Molecular Probes) and used as reference probes. Chromosomal locations of these probes were validated in metaphases of normal individual.

### Statistical analysis

All analyses were performed using the statistical software IBM SPSS Statistics Version 21 (Armonk, NY). Continuous variables were assessed by Pearson correlation analyses. The comparison of categorical variables was examined by Pearson chi-square test with Yates continuity correction. Difference was considered statistical significant if the *P* value was less than 0.05.

## Results

### GEP DNA copy number by QuMA

The GEP DNA copy number was measured by QuMA using real-time PCR. The efficiencies of the PCR were examined (Additional file 1: Figure S1). The CT values were plotted against the amount of DNA in serial dilutions. Both assays showed efficiencies close to 100%, demonstrated that the PCR products were nearly doubled in each cycle. The DNA copy number was calculated by the formula described in the Method section.

**Figure 1:**
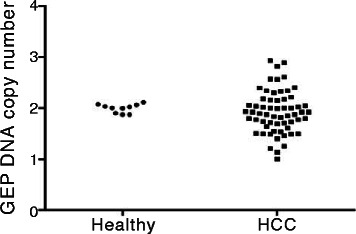
GEP DNA copy number determined by QuMA. Healthy blood DNA (n = 10) showed trivial variations on DNA copy number. Notably, HCC tumor DNA (n = 60) demonstrated considerable variations on GEP DNA copy number.

GEP DNA copy numbers were stable in the ten healthy individuals (DNA copy number ranged 1.88 to 2.12, SD = 0.09) (Figure 1). These measurements were used as reference for the diploid (N = 2) status, and with regard to the tolerance interval, GEP copy number >2.28 was considered significantly higher copy number than control. In HCC, GEP copy number variations were common (ranged 1.00 to 2.95, SD = 0.42) and 20% HCC (12/60) demonstrated gain of GEP DNA (Figure 1).

### Characterization of 17q21 region by FISH analysis

**Figure 2:**
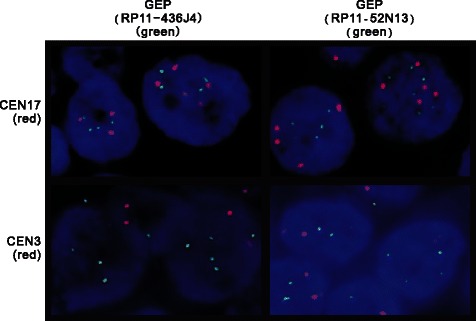
GEP gene copy number by FISH analysis with reference to centromere 17 (CEN17) and centromere 3 (CEN3). GEP gene (green) was detected by two flanking probes, RP11-436 J4 (left) and RP11-52 N13 (right), respectively. Control probes (red) included the centromere 17 (CEN17) and centromere 3 (CEN3). DNA copy number for each set was quantified for 100 cells and the scores (signals per cell) presented in Table 1. This case HCC801 showed CEN17 scores ranged 3.37 to 3.51, and GEP scores 3.66 to 3.80. The data indicated an increased chromosome 17 copy number at centromere and GEP locus at 17q21. Nonetheless, CEN3 scores ranged 1.97 to 2.17, indicating approximately two copies of chromosome 3 (diploid) with reference to GEP scores 3.73 by different probes flanking the gene region. GEP copy number for this case HCC801 was 3.60 by FISH analysis (reference to CEN3) compared to 2.56 by QuMA (reference to D3S1609). QuMA is a PCR-based assay method and the extend of underestimation would depend on the percentage of non-tumor cells, e.g. infiltrating lymphocytes etc., within the tumor mass. Details have also been described in Discussion.

To further substantiate the gene copy number by QuMA, we have examined the copy number of 17q21 region in primary HCC samples (HCC801 and HCC884) by FISH analysis. Both BAC clones (RP11-436 J4 and RP11-52 N13) flanking GEP gene demonstrated increased DNA copy number (Figure 2; Table 1). Nonetheless, the centromeric probe at chromosome 17 (pEZ17-4) also revealed increased DNA copy number per cell (Table 1). CEN17 scores ranged 2.92 to 3.51 per cell, and GEP scores ranged 3.02 to 3.80 per cell. The data indicated an increased chromosome 17 copy number, at both the centromere and GEP locus in these cases.

**Table 1:** **GEP DNA copy number with reference to A.) centromere 17 (CEN17)** ^**1**^ **and B.) centromere 3 (CEN3)** ^**2**^ **by FISH analysis** ^**3,4**^

A.	GEP	CEN17	GEP	CEN17
HCC	RP11-436 J4	pEZ17-4	RP11-52 N13	pEZ17-4
801	3.66	3.37	3.80	3.51
884	3.08	2.92	3.02	2.77
**B.**	**GEP**	**CEN3**	**GEP**	**CEN3**
**HCC**	**RP11-436 J4**	**pAE0.68**	**RP11-52 N13**	**pAE0.68**
801	3.73	2.17	3.73	1.97
884	2.85	2.10	2.89	1.92

^1^GEP DNA copy number with reference to centromere 17 (CEN17). In these two cases, CEN17 scores ranged 2.92 to 3.51 per cell, and similarly GEP scores ranged 3.02 to 3.80 per cell. The data indicated an increased chromosome 17 copy number, at centromere and GEP locus at 17q21, in these cases.

^2^GEP DNA copy number with reference to centromere 3 (CEN3). CEN3 scores ranged 1.92-2.17 per cell, indicating approximately two copies of chromosome 3. The number of GEP DNA per 2 centromere (diploid) was 3.60 and 2.86 for HCC801 and HCC884, respectively, by FISH analysis. GEP copy number was 2.56 and 2.93 for HCC801 and HCC884, respectively, by QuMA (reference to D3S1609).

^3^DNA copy number for each set was quantified for 100 cells and data presented per cell.

^4^Noted independent platform using QuMA qPCR, both HCC801 and HCC884 showed similar increased GEP DNA copy number.

To further comprehend the GEP DNA copy number, we therefore used centromere 3 as reference chromosome for FISH analysis as chromosome 3 as shown to be stable on copy number [19,20,22]. CEN3 scores ranged 1.92 - 2.17 per cell, indicating approximately two copies of chromosome 3 in these cases. The number of GEP DNA per 2 centromere (diploid) was 3.60 and 2.86 for HCC801 and HCC884, respectively, by FISH analysis with reference to CEN3. Notably, similar GEP DNA copy number increased was observed in both HCC801 and HCC884 using QuMA. The data indicated chromosomal gain of the GEP gene locus at 17q21.

### GEP copy number correlated with transcript levels and clinico-pathological features

**Figure 3:**
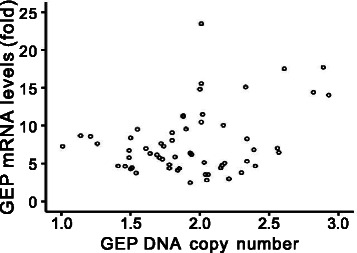
GEP DNA copy number correlated with expression levels. GEP DNA copy number correlated with transcript levels (n = 60, *r* = 0.331, *P* = 0.010).

GEP transcript levels had been demonstrated to be significantly elevated in HCC compared with their adjacent non-tumor liver tissues and normal livers from healthy individuals [8,15]. The transcript data was extracted from the previous reported cohort [8] and analyzed with the current DNA data. Notably, GEP DNA copy number correlated with transcript levels (n = 60, *r* = 0.331, *P* = 0.010) (Figure 3). Importantly, in HCC cases with GEP gene amplification, increased GEP gene copy number was tightly associated with enhanced expression levels (n = 12, *r* = 0.664, *P* = 0.019).

The GEP copy number in HCCs were further analyzed for clinico-pathological significance. The HCCs were categorized as “no gain” or “gain” groups according to the GEP DNA copy number. GEP DNA copy number was significantly associated with HBV status (*P* = 0.015) (Table 2).

**Table 2:** **Clinico-pathological features of HCC in relation to GEP DNA copy number**

	GEP DNA copy number		
HCC features	No gain	Gain	*P* ^1^
Venous infiltration			
Absent	19	7	0.397
Present	29	5	
Tumour size			
Small (≤5 cm)	10	3	1.000
Large (>5 cm)	38	9	
pTNM stage			
Early stage (I-II)	31	9	0.732
Late stage (III-IV)	17	3	
Edmondson grade			
Well differentiation	41	10	1.000
Poor differentiation	7	2	
Gender			
Male	42	7	0.055
Female	6	5	
Age			
Young (≤60)	37	6	0.133
Elderly (>60)	11	6	
Serum AFP level			
Low (≤20 ng/mL)	21	1	0.052
High (>20 ng/mL)	27	11	
HBV status (HBsAg)			
Negative	4	5	0.015
Positive	44	7	

^1^Pearson chi-square test with Yates continuity correction.

*Abbreviations*: *AFP* alpha-fetoprotein, *HBV* hepatitis B virus, *HBsAg* hepatitis B surface antigen.

## Discussions

Increased GEP transcript and protein levels have been reported in various human cancers [5-7]. The enhanced GEP expressions have been demonstrated to associate with aggressive tumor features, including large tumor size [15,24], metastasis [15,25], and poor prognosis [5,8,25-27]. Biological roles have been demonstrated with cell models and xenograft systems with regulatory functions on growth [7,15,25,28,29], invasion [15,30,31], tumorigenicity [15,25,30], drug resistance [8,28,30,32] and cancer stem cell properties [8,9]. The biological function of GEP corroborates very well with the aggressive clinical features of the tumors showing over-expression. Studies on the signaling pathways demonstrated substantial molecules associated with GEP expressions. GEP stimulated MAPK and PI3K pathways [29]. GEP was a cofactor for toll-like receptor 9 signaling [33]. In addition, GEP protein over-expressions were associated with accumulation of wild-type p53 protein [34]. GEP has also been shown to be regulated by endothelin, lysophosphatidic acid and cAMP [7]. Protein kinase C signaling has demonstrated to influence GEP protein levels [35]. Nonetheless, the exact mechanism for GEP over-expression in the majority of human cancers warrant further investigation.

Elevated expressions of growth factors and oncogenes have been reported to associate with gene copy number gain. In the current study, the GEP gene copy number was investigated by real-time PCR based method QuMA and FISH analysis. QuMA has been commonly employed for quantitative measurement of DNA copy number [21,36,37]. Nonetheless, there were technical limitations that DNA samples were extracted from tumor mass which would contain tumor cells and tumor-infiltrating cells including lymphocytes etc. Therefore, copy number assays would underestimate the tumor chromosomal aberrations and would need further computational analyses [38,39]. The extent of underestimation would depend on the percentage of tumor-infiltrating cells which fluctuate between specimens. In contrast, FISH analysis was performed under microscope. Thus, tumor cells could be distinguished from non-tumor components and focused to analyze for genetic alterations. FISH has been shown to be useful to facilitate the diagnosis of neoplasms [40-42]. Comparatively, FISH is reliable but technically demanding and expensive, while QuMA prone to underestimate genetic aberration but is economical and suitable for large scale screening by automation. The two methods should be used in complementary for investigation of cancer genetic aberrations.

Copy number gain at specific chromosomal region contributes on activation of oncogenes and growth factors. The process is important during tumor initiation and also progression along carcinogenesis. However, there always have concerns on whether high levels of amplification are necessary or if the gain of single extra copy would be able to advance cancer. Recently, there are reports that low copy number gain contributes on cancer progression [43-45]. Gain of single supernumerary segment encompassing Myc, Pvt1, Ccdc26 and Gsdmc has shown to promote cancer [43]. The present study demonstrated low copy number gain at centromere 17 and GEP gene at 17q21 associated with increased GEP expressions. Therefore, the specific chromosomal region 17q21 would be the focus to examine if this segment contains the essential gene set for tumor initiation and progression.

Recurrent genomic and expression alterations have been reported on chromosomal arm 17q. Independent studies demonstrated expression gains of gene set at 17q12-21 [46] and 17q21-25 [47], respectively, in HCC by expression imbalance map analysis. These expression gain regions corroborated with chromosomal gain regions frequently reported in HCC [48,49]. Furthermore, TOP2A gene locus at 17q21-22 has reported copy gains and overexpression, and regulated chemo-resistance in HCC [47]. Similar copy number gain status has also been revealed in HER2 with copy number gain at gene locus 17q21 and centromere 17, and showed protein over-expression in breast cancer [50]. Elevated HER2 expression has been demonstrated in HCC tissues [51] and blood samples [52], and associated with poor survival [49]. HER2 associated with hepatitis B virus infection [52] in particular hepatitis B x (HBx) antigen [51,53] where HBx has been shown to promote chronic liver disease and HCC development [54]. Further investigation would be warranted to examine the minimal gene set that drives neoplasia. Potential candidates at chromosomal segment 17q21 that demonstrated copy number gain and overexpression included GEP, TOP2A and HER2. These genes could constitute partly the essential gene set that initiate and promote HCC progression.

## Conclusion

These observations show copy number gain of GEP gene at 17q21 in 20% HCC, and the increased GEP gene copy number correlated with enhanced expression levels in these HCC. This partly provides a mechanistic explanation for the over-expression of GEP for the subset of HCC. Future studies should also examine the chromosomal region at 17q21 for the minimal essential set of genes for HCC initiation and progression. Notably, GEP over-expression has been observed in over 70% HCC [8,15]. Further investigations are warranted to understand tumor that showed GEP over-expression in the absence of GEP gene copy number gain.

## Additional file

Additional file 1: Figure S1. PCR efficiencies. Serial dilutions of template DNA was used for the PCR. The CT values were plotted against the amount of DNA used. Both assays showed efficiencies close to 100%, denoted that the PCR products were amplified with a factor close to 2 in each cycle.

## Abbreviations

GEP: Granulin-epithelin precursor
HCC: Hepatocellular carcinoma
