# Supplementary material for: Copy number gain of granulin-epithelin precursor (GEP) at chromosome 17q21 associates with overexpression in human liver cancer
Source: BMC Cancer. 2015 Apr 11;15:264. doi: 10.1186/s12885-015-1294-x (PMC4403714; doi:10.1186/s12885-015-1294-x)
Supplement: Additional file 1: Figure S1. — PCR efficiencies. Serial dilutions of template DNA was used for the PCR. The CT values were plotted against the amount of DNA used. Both assays showed efficiencies close to 100%, denoted that the PCR products were amplified with a factor close to 2 in each cycle. [file 12885_2015_1294_MOESM1_ESM.pdf]

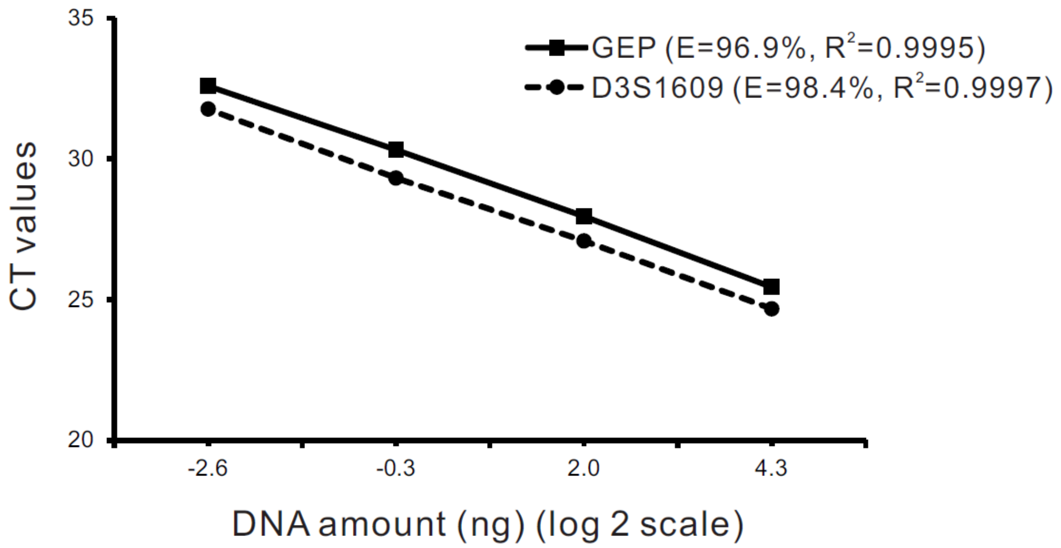

Supplementary Figure 1. PCR efficiencies. Serial dilutions of template DNA was used for the PCR. The CT values were plotted against the amount of DNA used. Both assays showed efficiencies close to 100%, denoted that the PCR products were amplified with a factor close to 2 in each cycle.
